# A spindle cell carcinoma presenting with osseous metaplasia in the gingiva: a case report with immunohistochemical analysis

**DOI:** 10.1186/1746-160X-4-28

**Published:** 2008-12-01

**Authors:** Naoki Katase, Ryo Tamamura, Mehmet Gunduz, Jun Murakami, Jun-Ichi Asaumi, Goichi Tsukamoto, Akira Sasaki, Hitoshi Nagatsuka

**Affiliations:** 1 Department of Oral Pathology and Medicine, Graduate School of Medicine, Dentistry and Pharmaceutical Sciences, Okayama University, Okayama, 700-8525, Japan; 2 Department of Oral and Maxillofacial Radiology, Graduate School of Medicine, Dentistry and Pharmaceutical Sciences, Okayama University, Okayama, 700-8525, Japan; 3 Department of Oral and Maxillofacial Surgery and Biopathological Science, Graduate School of Medicine, Dentistry and Pharmaceutical Sciences, Okayama University, Okayama, 700-8525, Japan

## Abstract

**Background:**

Spindle cell carcinoma (SpCC) is a rare, high malignant variant of squamous cell carcinoma (SCC), which shows biphasic proliferation of conventional SCC component and malignant spindle shape cells with sarcomatous appearance.

**Methods:**

A case of Spindle cell carcinoma with bone-like calcified materials, occurring at the mandibular molar region of 71-years-old Japanese male patient was presented with gross finding, histological findings and MRI image. To identify the characteristics of the bone-like materials, immunohistochemistry were performed.

**Results:**

Histologically, the cancer cells were composed of spindle cells and epithelial cells which form nests with prominent keratinization. Histological findings showed typical histology of the SpCC, however, as an uncommon finding, spatters of calcified, bone-like materials were observed in between the cancer cells. Immunohistochemistry revealed that cancer cells were positive for cytokeratins and vimentin to a varying degree and negative for Desmin, S-100, Osteopontin, BMP-2 or BMP-4. These findings implied that the calcified materials were formed by metaplasia of the stromal cells.

**Discussion:**

Bone-like materials formation by osseous and/or cartilaginous metaplasia of the stroma in the carcinoma has been reported. However, the detailed mechanism of these metaplasia and affection on the clinical feature, prognosis and therapies are not well established. In summary, we presented an unique case of SpCC, which has not been described in the literature.

## Background

Spindle cell carcinoma (SpCC), also known as sarcomatoid carcinoma or pseudosarcoma, of head and neck is a rare neoplasm. SpCC is known as a high malignant variant of squamous cell carcinoma, which is composed of conventional squamous cell carcinoma component, either in-situ and/or invasive and malignant spindle component with sarcomatous appearance. Although it is generally accepted that SpCC is a monoclonal epithelial neoplasm [1-5], and the sarcomatous components are derived from squamous epithelium with divergent mesenchymal differentiation [6], the diagnosis, classification and management of this tumor infrequently may become subject matter deluded of its histological variety in sarcomatous components. These sarcomatous components commonly resemble to fibrosarcoma or malignant histiocytoma [7,8], and while rare, foci resembling to chondrosarcoma and/or osteosarcima differentiation may be observed [9].

This is a case report of a spindle cell carcinoma of gingival mucosa presenting with bone-like calcified materials.

## Case presentation

A 71-year-old Japanese male patient was referred to the Okayama University Hospital, complaining the swelling at the left side of the mandibular molar region. The lesion was 44 × 29 mm in size, exophytic with rough and irregular surface. The patient's medical and family histories were unremarkable.

**Figure 1:**
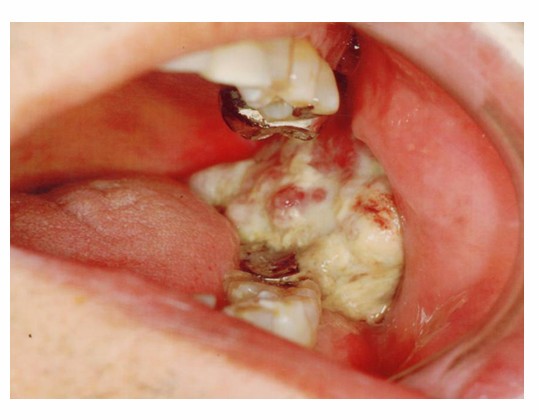
**Gross finding of the tumor**. Intraoral examination showed exophytic, polypoid mass with irregular surface in the mandibular molar region.

**Figure 2:**
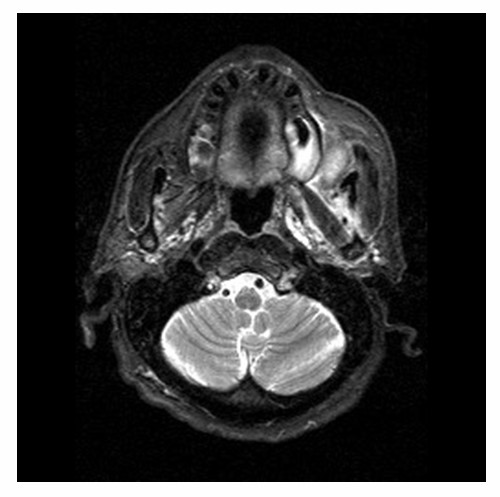
**Axial MRI imaging of the tumor**. The margin of the lesion was clear, no invasion was observed.

**Figure 3:**
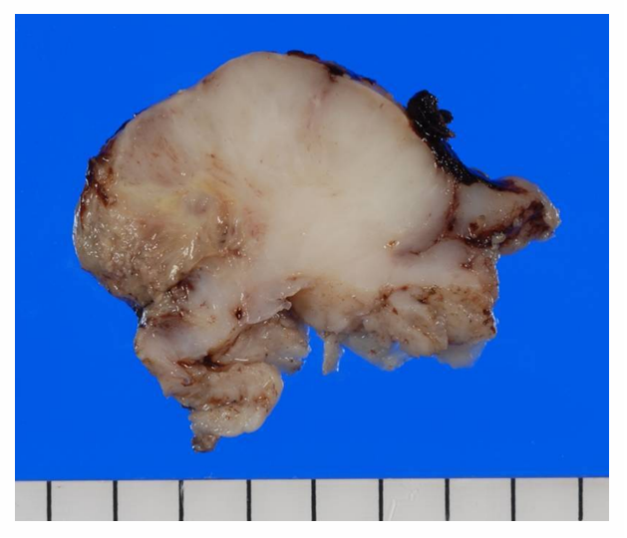
**Macroscopic image of the tumor**. The lesion was a fragile mass with ulcerated surface. The cut surface was grayish-white in color, myxoid or lobular pattern in some areas.

Clinically, the lesion was elastic soft and fragile, and the surface of the lesion was ulcerated [Fig. 1]. The margin of the lesion was clear, and MRI scan revealed no invasion into circumjacent tissues [Fig. 2]. Moreover, invasion into the mandibular bone was not prominent on the CT images. With the suspicion of poorly differentiated squamous cell carcinoma from the biopsy, surgical resection with neck dissection was performed. The lesion was a fragile mass, grayish-white in color [Fig. 3].

No tumor cells were observed around the surgical margin, and lymph node metastasis was not observed. No recurrence or metastasis was detected since surgery so far.

**Figure 4:**
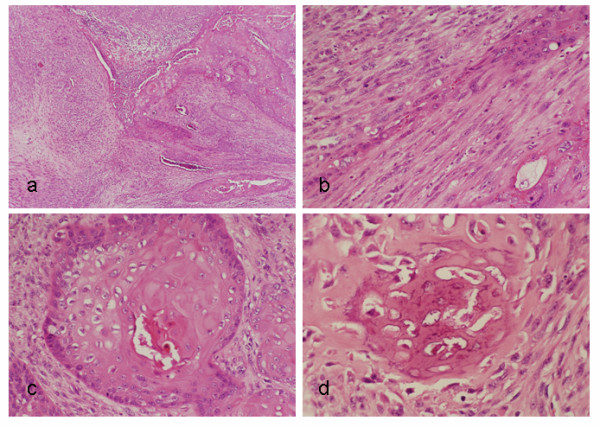
**Histological findings**. (a) The cancer cells were composed of spindle cell component and epithelial component. (b) Transition of spindle cells into epithelial component was observed. (c) Conventional squamous cell carcinoma components presenting with keratinization were also observed. (d) Spatters of calcified materials with bone-like appearance were observed.

Histologically, the tumor showed biphasic appearance. The bulk of the tumor was composed of invasive, spindle shape cells, which arranged irregularly with bundle formation resembling to fibrosarcoma. Together with the spindle shape tumor cells, proliferation of polygonal epithelial cells were observed, forming tumor nests with distinct keratinization similar to cancer nest observed in well differentiated squamous cell carcinoma. In some areas, spindle shape cells were transitional to the epithelial cancer nest-like structure. The stromal cells were intermingled with the spindle shape cells, showing myxoid appearance. In some areas, spatters of calcified, bone-like materials were observed in between the malignant spindle cells, which showed osteosarcimatous appearance [Fig. 4]. From all these histological findings, the lesion was diagnosed as spindle cell carcinoma (SpCC) with osteosarcomatous differentiation.

To confirm the diagnosis, immunohistochemistry was performed according to avidin-biotin-peroxidase complex (ABC) method. The detail of the antibodies used for immunohistochemistry is summarized in Table 1.

**Table 1:** Antibodies used for the present case

**Antibody**	**Source**	**Result**	
		**Spindle cells**	**Epithelial cells**
Cytokeratin AE1/AE3	DAKO(USA)	+	+
Cytokeratin 8	Roche Diagnostics (Germany)	+/-	+
Cytokeratin 19	Novocastra (UK)	+/-	+
Vimentin	DAKO(USA)	+	-
Desmin	DAKO(USA)	-	-
S-100	Nichirei (Japan)	-	-
αSMA	DAKO(USA)	+/-	+/-
Osteopontin	IBL (Japan)	-	-
BMP-2	Santa Cruz (USA)	-	-
BMP-4	Santa Cruz (USA)	-	-
Ki-67	DAKO (USA)	<30%	

αSMA: alpha smooth muscle actin, BMP: bone morphogenetic protein

+: positive, +/-: focally positive, -: negative

**Figure 5:**
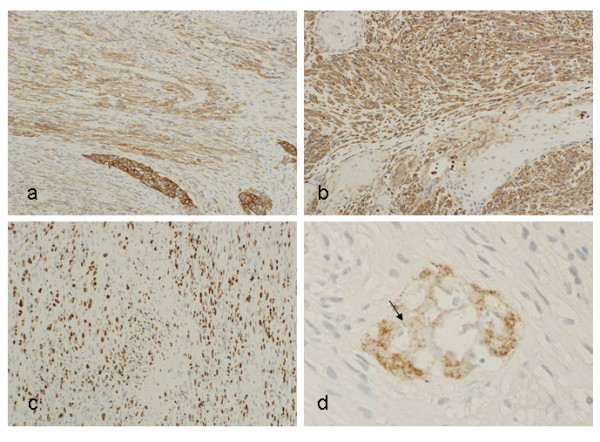
**Immunohistochemistry**. (a) Cytokeratin AE1/AE3 was positive both in spindle shape cells and epithelial nests. (b) Vimentin was only positive in spindle shape cell component, while it is negative in the epithelial nests. (c) Ki-67 index of the cancer cells was >30%. (d) The matrix of the calcified materials (arrows) showed positive reaction for osteopontin, while cancer cells did not.

Immunohistochemistry revealed that spindle shape cells showed positive reaction for cytokeratins, vimentin, negative reaction for S-100 protein and desmin, squamous cell carcinoma-like nest forming epithelial components showed positive reaction for cytokeratins, both low- and high-molecular weight, negative for vimentin, S-100 and desmin. Alpha smooth muscle actin (αSMA) was vaguely positive in both spindle shape cells and epithelial cells. The cells around bone-like calcified materials showed positive reaction for low-molecular weight cytokeratin and vimentin, osteopontin was only positive in the bone matrix-like area. Cancer cells did not show positive reaction for osteopontin, bone morphogenetic protein (BMP) -2 or BMP-4. Ki-67 index was around 30% at a maximum, but that of the cells around bone-like materials was lower [Fig. 5]. Thus the bone-like materials were considered as metaplastic bone formation.

## Discussion

SpCC is a rare variant of squamous cell carcinoma. Its most frequently affected sites is larynx, however, it may infrequently occur in various organs; gingiva [2,10], tongue [11,12], upper aerodigestive tract including hypopharynx and nasal cavity [6,13,14], esophagus, skin and breast [15].

It is generally understood that the diagnosis of SpCC requires the demonstration of both components [16]. In accord with this criterion, typical histology of SpCC was observed in the present case, which was composed of conventional squamous cell carcinoma component and spindle shape cells with sarcomatous appearance.

However, the existence of bone-like calcified materials was the uncommon, controversial finding for SpCC in oral mucosa. To the best of our knowledge, this finding was not described in previous report. In the immunohistochemical examination, both epithelial component and spindle shape cell component showed positive reaction for cytokeratins to a varying degree, but vimentin positive cells were limited to the spindle shape cell component. These results were consistent to the previous reports [1,14,17-19]. The cells around the materials were vimentin-positive low activity cells, and cancer cells did not show positive reaction for neither BMP-2 nor BMP-4, which indicates these materials were formed by mesenchymal metaplasia of the stromal cells, but not by the tumor cells itself.

Although the incidence of osseous metaplasia is rare finding in oral SpCC, it is occasionally observed in that of larynx [8]. While also rare, the formation of bone-like and/or cartilage-like materials by the metaplasia of stromal cells is often reported in various kind of carcinomas, such as laryngeal cancer [20], esophageal cancer [21,22], colon cancer [23-25], lung cancer [26], and breast cancer [27]. The histogenesis of the osseous and/or cartilaginous metaplasia in the carcinoma has been come up to debate so far. The biological mechanism of these metaplastic changes is considered to be caused by stromal activation associated with human-host interface [20]. Although some report implies the formation of these materials is related to radiation therapy [21], the mechanism of stromal metaplasia is still unclear. Moreover, the affection of these metaplastic bone or cartilage on the clinical features, prognosis, and response to radiation therapy or chemotherapy are not well established.

## Conclusion

In summary, we reported a unique of SpCC with calcified bone-like materials in the gingiva, which is a very rare case in the literature.

## Consent

Written informed consent was obtained from the patient for publication of this case report and any accompanying images. A copy of the written consent is available for review by the Editor-in-Chief of this journal.

## Abbreviations

SpCC: Spindle cell carcinoma

## Competing interests

The authors declare that they have no competing interests.

## Authors' contributions

NK, HN and RT carried out the case study, discussed and reviewed the literature and prepare the manuscript. GT and JM contributed to the collection of clinical and/or radiological data and discussion. GM, JA and AS participated in review process and carried out critical revision of the manuscript.
